# A Pilot Study for Forgiveness Intervention in Adolescents With High Trait Anger: Enhancing Empathy and Harmony

**DOI:** 10.3389/fpsyg.2020.569134

**Published:** 2020-12-23

**Authors:** Linjin Tao, Mingxia Ji, Tingting Zhu, Hong Fu, Ruoying Sun

**Affiliations:** ^1^School of Psychology, Nanjing Normal University, Nanjing, China; ^2^Guangming Branch of Shenzhen Institute of Education Sciences, Shenzhen, China; ^3^Institute of Medical Humanities, Nanjing Medical University, Nanjing, China; ^4^Personnel Department, Suzhou Vocational Institute of Industrial Technology, Suzhou, China

**Keywords:** forgiveness intervention, trait anger, adolescent, empathy, harmony, juvenile delinquents

## Abstract

Forgiveness interventions benefit victims’ mental health, reduce levels of anger, and promote forgiveness. However, forgiveness interventions are rarely used to improve the offender’s anger and mental health, especially in specific situations such as juvenile correctional facilities. The offender is often also a victim, and reducing the offender’s excessive anger may prevent or decrease the likelihood of future interpersonal violence. This study examined the effects of forgiveness interventions on anger, forgiveness, empathy, and harmony of juvenile delinquents with high levels of trait anger. Eighteen adolescents with trait anger in a juvenile correctional facility volunteered to participate in group counseling. A pretest–posttest method of quasi-experimental design was used, with 8 participants in the intervention group and 10 in the control group; the intervention group received forgiveness group counseling, and the control group did not. The results revealed that the intervention group had significantly higher scores for forgiveness, empathy, and harmony than the control group, although no significant differences in the scores of state and trait anger were found. The forgiveness intervention had significantly improved the levels of forgiveness toward specific perpetrators of childhood victimization for the juvenile delinquents with high levels of trait anger, raising their levels of empathy and harmony; there was no significant increase in trait anger. The findings indicated that forgiveness intervention provides an effective way to improve the positive mental strength of adolescents with high levels of trait anger.

## Introduction

Agnew’s general strain theory (GST) posits that crime is a consequence of “negative relationships with others” or strain (Agnew, 1992; Jang and Agnew, 2015). GST defines strains as events and conditions that are disliked. Those specific strains most conducive to crime are high in magnitude, are seen as unjust, are associated with low social control, and provide some pressure or incentive for crime. Examples include parental rejection; harsh, erratic parental discipline; child abuse and neglect; and negative secondary school experiences (Sigfusdottir et al., 2012). Agnew proposes that strain generates negative effective states, such as anger and frustration, which create pressure for corrective action, including crime. For Agnew, anger is the “most critical emotional reaction for the purpose of the general strain theory” and is said to energize individuals for action, reduce concern for the consequences of one’s behavior, and create a desire for revenge. Anger has been identified as a basic, primary emotion that leads to violence and aggression (Siever, 2008). People with a stable and context-irrelevant tendency to experience anger are easily provoked by a variety of situations; such persons are referred to as individuals with “trait anger” (Spielberger et al., 1983) (i.e., “high-trait-anger individuals”). Adolescents with high trait anger are often accompanied with psychological and behavioral problems such as anxiety, depression, and violence, which bring potential risks to themselves, families, and society.

Trait anger has a significantly positive correlation with negative life events (Puskar et al., 2008), as frustration and a sense of injustice caused by negative life events are important triggers of anger (Potegal and Stemmler, 2010). Recent studies have identified that multiplicity and severity of victimization exposure in a prison sample were positively associated with chronic anger (Erzar et al., 2018). The relationship between early victimization, violent behavior, and crime has been supported by several studies, and anger plays an important role in this relationship. Studies of juvenile delinquents have found that juvenile offenders experience more trauma, are less supported by their families, receive less schooling, and are less able to cope with social challenges and stress than their non-offending peers (Dierkhising et al., 2013; Baglivio et al., 2014). Additionally, adverse childhood experiences relate to repeat offenses by juvenile offenders (Wolff et al., 2017). Therefore, Reavis et al. (2013) suggested that attention should be paid to the influence of early life experience in the treatment and intervention of criminals. However, few studies have explored how to help criminals identify and cope with stressors in life and deal with past injuries again (Toma et al., 2018). Some researchers believe that if we can define our clinical work more broadly, it may help us to identify the sources of frustration and anger, especially to promote the process of family repair of hatred and dissatisfaction. In this way, not only can individuals “manage” their anger, but also more importantly, they will not be so angry from the beginning (Barish, 2009).

However, frustration does not necessarily lead to stable anger or aggression. According to the integrative cognitive model proposed, individual differences in three cognitive processes jointly contribute to a person’s level of trait anger and reactive aggression (Wilkowski et al., 2010): (1) “an automatic tendency to attribute hostile traits to others” (Wilkowski et al., 2007); (2) “rumination on hostile thoughts”; (3) “effortful control” (Wilkowski et al., 2010). The key mechanism in the process of effortful control in regulating anger and aggressive behavior is forgiveness (Wilkowski and Robinson, 2010), which is the process in which, after the transgression, the victim’s negative cognitive, affective, and behavioral reactions toward the offenders gradually disappear and are replaced by positive cognitive, affective, and behavioral reactions (Enright et al., 1989). Forgiveness also includes the process of a victim undergoing a series of prosocial intent changes so as to feel empathy for the offender (McCullough et al., 1998). Forgiveness intervention (FI) has been scientifically demonstrated to decrease excessive anger in victims (Enright and Fitzgibbons, 2015), but there is still a need to test whether FIs have an effect on trait anger in adolescents who are victimized early in their lives. This study attempts to use FI to help trait-anger adolescents in juvenile facilities successfully engage in the process of forgiving early offenders in order to reduce their trait-anger levels.

According to positive psychology theory, an effective approach to dealing with a problem is to help individuals find resources that will aid them in becoming healthier and happier instead of focusing on the problem. Psychological harmony is an important aspect of mental health (Nie et al., 2015). The harmony of one’s mental state and interpersonal relationships is important for achieving psychological harmony. Moreover, the quality of one’s interpersonal relationships is a particularly important sign of mental health. Empathy facilitates satisfactory interpersonal contact (Carnicer and Calderon, 2014). With a high level of anger, a lack of empathetic responsiveness toward others has also been identified as an antecedent to aggressive behavior (Day et al., 2012). Empathy is the capacity to understand or feel what another person is thinking and experiencing within that person’s frame of reference. Individuals who tend to perceive ambiguous situations as hostile (such as trait-anger adolescents) often lack the capacity to place themselves in another’s position. Meta-analysis has found that FIs can produce significant positive effects, including satisfaction, happiness, confidence, hopefulness, energy, soft-heartedness, warmth, and compassionate (Akhtar and Barlow, 2018). Therefore, if FI can increase the level of empathy and mental harmony, it may be an effective method of psychological construction. This study aims to explore whether we can establish a positive psychological construction for trait-anger adolescents in juvenile facilities to increase their positive mental strength so that they might confront potential risks in life with more resources.

Given the above background, this study focused on the negative life events and offensive experiences of trait-anger adolescents, employing FI to this end. We screened 18 adolescents with high trait anger from the juvenile delinquency center, where such kind of adolescents relatively concentrated.

From the perspective of restorative justice (RJ), FI is also the proper meaning of RJ to intervene in the source of these crimes. RJ is an approach to criminal justice that considers crime an act of harm committed by a perpetrator against an individual or community (Lloyd and Borrill, 2020). This interpersonal transgression creates an obligation for the offender to repair the damage done by such an act and restore the stakeholders to their prior status (Zehr, 1990). For the juvenile delinquents, there are many possibilities for their future, and it is often difficult to recover the interpersonal injuries caused by crimes. However, it is often overlooked what kind of people they will become in the future and whether they will continue to cause harm to society and others because of their unfinished events. Forgiving intervention can work in this area. Through engaging in restorative activities, it is suggested that the individual comes to redefine himself/herself as a law-abider and subsequently no longer engages in criminal activity (Tyler et al., 2007). In addition, trait anger may be used as an indicator of identification. It is more socially meaningful to intervene when adolescents have not committed illegal behaviors but have such tendencies.

The research hypotheses were that—for adolescents with high trait anger—FI (1) will increase the levels of forgiveness toward a particular offender, (2) improve the levels of empathy and harmony, and (3) decrease the levels of trait anger.

## Materials and Methods

### Participants

This study combined random sampling with cluster sampling. The subjects for the study were 180 volunteers (male = 160, female = 20) from a juvenile correctional facility who participated in an assessment to find individuals who had been severely victimized and who also had high levels of trait anger. An offense event questionnaire (recalling and describing a specific offender and offending event), the 12-item form of the Transgression-Related Interpersonal Motivations Scale (TRIM-12; McCullough et al., 1998), and the Trait Anger Scale (TAS; Spielberger et al., 1983) were administered to the 18 participants who met both conditions above and who were willing to participate in group counseling. Participants’ ages ranged from 16 to 19 years (mean = 17.50, *SD* = 0.924), the majority were male [13 (72.22%)]. On average, they had 7.61 (*SD* = 1.54, range = 5–11) years of education. Taking into consideration the factors of age, consistency of trait-anger scores, sex ratio, group size, and group counseling settings (the content of the consultation arrangement required each group to have an even number of members), we assigned 8 of the 18 adolescents to the intervention group and the remaining 10 to the control group. There was no significant difference in trait anger of pretest [*U*(8, 10) = 39.50, *p* > 0.05]. The group structure and demographic information are shown in Table 1.

**TABLE 1:** Group structure of the intervention and control groups.

		Level of education (n)			Gender (n)		Age (n)				Region (n)		Trait anger (mean rank)

		Primary School	Middle School	High School	Male	Female	16	17	18	19	Urban	Rural	
Intervention		2	5	1	6	2	1	4	2	1	1	7	9.44
Control		2	8	0	7	3	1	4	3	2	2	8	9.55
Chi-square tests	χ^2^	1.49			0.06		0.32				0.18		*U*-Test *U* = 39.50 *p* > 0.05
	*p*	*p* > 0.05			*p* > 0.05		*p* > 0.05				*p* > 0.05		

### Experimental Procedure

Nanjing Normal University Ethics Committee approval was received. Confidentiality of the participant was ensured in several ways, i.e., name of all participants was anonymized, and all recordings were kept confidentially. Twenty-minute intake interviews were conducted with all 18 participants. The questions mainly focused on (1) whether the participant believed he/she became angry easily, (2) whether people around the participant (relatives, friends, etc.) considered him/her easily angered, and (3) whether there was any connection between the offensive experience described in the initial screening questionnaire and the participant’s tendency to get angry as a personality trait.

This study applied a pretest–posttest quasi-experimental design: the participants in the intervention group received forgiveness counseling, and those in the control group participated only in their regular work and activities. Participants in both groups were administered the same psychological tests before and after the intervention. The experimental effects of the intervention are shown in Table 2. Considering the limitation of testing only state anger before and after the intervention, after each intervention session, the participants in the intervention group were given the State Anger Scale (SAS) (because of constraints, the control group was not monitored) to complete at 20:00 every day so as to observe the dynamic changes of the state anger.

**TABLE 2:** Pretest-posttest of quasi experimental design with the intervention and control groups.

Group	Pretest	Intervention program	Posttest	Comparison
Intervention	Test (m1)	Forgiveness group counseling, twice a week, 120 min each session for consecutively 14 times	Test (m3)	M1 = m3–m1
Control	Test (m2)	No intervention	Test (m4)	M2 = m4–m2

*The experimental effects of intervention were revealed by the Mann-Whitney U-test of the difference between M1 and M2.*

### Instruments

#### The FI Program

This study’s intervention program was based on the framework of the Enright Forgiveness Intervention Model (Enright, 2001; Knutson et al., 2008), which emphasizes four key phases: (1) the uncovering phase, in which the individual confronts the nature of the offense and uncovers the consequences of having been offended; (2) the decision phase, in which one makes a decision to commit to forgiveness; (3) the work phase, in which one actually works on forgiving and practices empathy and compassion for the offender; and (4) the deepening phase, in which one deepens one’s will and ability to forgive, overcoming obstacles standing in the way of forgiveness. Following the basic group counseling principles, such as group dynamics theory, and combining specific counseling theories (cognitive reconstructing, etc.) and positive psychology conceptions, we designed the preliminary intervention group program for forgiveness. We then performed an expert validity test, integrating opinions collected from five experts to refine the intervention program. The new designs for the intervention program were presented to experts until there were no more suggestions. The final design included 14 sessions of group counseling in six units, with each session lasting 2 h and sessions being held twice a week. Researchers interested in this intervention should contact the corresponding author.

#### Assessment Scales

##### Forgiveness scales

The revised Enright Forgiveness Inventory (EFI) was designed to assess a subject’s level of forgiveness toward the offender (Subkoviak et al., 1995). The revised Chinese version consists of 50 items with six factors: positive affect, positive cognition, negative affect (NA), negative cognition (NC), negative behavior–avoidance (NB), and positive behavior (PB) (Tao, 2011). The EFI is a six-point Likert inventory with scores ranging from 50 to 300. The higher the score, the higher the level of forgiveness. The Cronbach’s coefficient for this scale was 0.98. The revised Chinese version of the TRIM-12 includes 12 items (Chen and Zhu, 2006) and two factors—revenge-seeking behavior and avoidance—and is measured on a five-point Likert inventory, with total scores ranging from 0 to 48. The higher the score, the lower the level of forgiveness. Cronbach’s coefficient for this scale was 0.87.

##### Anger scale

Spielberger’s State-Trait Anger Expression Inventory-2 was designed to assess state and trait anger (STAXI-2; Spielberger, 2010). The revised Chinese version of STAXI-2 includes two scales (Tao, 2011). The TAS assesses an individual’s frequency of experiencing anger (for example, “I have a hot temper”), including 10 items and two factors: angry temperament and angry reaction. The questions are measured on a four-point Likert scale (1 = rarely true, 4 = always true), with total scores ranging from 10 to 40. A higher score represents a stronger tendency to be angry. The SAS assesses the intensity of anger as an emotional state at a particular time (for example, “I feel angry”), including 10 items with three factors: anger affect, speech/action, and anger unleash. Responses are given on a four-point Likert scale ranging from 10 to 40. The SAS was used as a dynamic evaluation tool, and the participants were asked to complete the questionnaire at 20:00 every evening to continuously evaluate their state anger.

##### Harmony scale

A subscale of the Chinese Personality Assessment Inventory for Adolescents (CPAI-A) was jointly established by the Chinese University of Hong Kong and the Chinese Academy of Sciences Institute of Psychology. This scale includes 14 items and uses binary scoring (0 = false, 1 = true), for a total score ranging from 0 to 14. It is designed to assess the factor of harmony in personality, with a higher score meaning more harmony in personality. The average of the CPAI-A Cronbach’s was 0.72 (Cheung and Fan, 2008).

##### Empathy scale

The Chinese version of the Interpersonal Reactivity Index–Chinese (IRI-C) was revised by Wu Jingjie from Davis’s Interpersonal Reactivity Index (Davis, 1983; Wei, 2007) and includes 22 items with responses measured on a five-point Likert scale. This study used the “perspective taking” subscale to assess cognitive empathy and measured emotional empathy with the “compassionate care” subscale. The Cronbach’s alpha of the IRI-C was between 0.53 and 0.78. We also applied the Interpersonal Sensitivity scale (from CPAI-A, the same scale applied in the previous paragraph as the “Harmony Scale”) to test individuals’ sensitivity to others’ thoughts and feelings. This scale included 12 items and used binary scoring ranging from 0 to 12. The higher the score, the more sensitive the individual.

#### Statistical Processing

The normal distribution test showed that the data in this study did not conform to the normal distribution, while the non-parametric test was applicable. As rank sum test of two independent samples, Mann–Whitney U tests were performed to identify if there were significant differences between the two groups (the intervention group versus control group) for high-trait-anger adolescents in regard to their ordinal scores in the changes of forgiveness, harmony, empathy, state anger, and trait anger before and after the FI and calculated the effect value. The effect value is an index of statistical efficacy used to measure the intensity of the intervention effect in intervention research (Durlak, 2009). In a study with a sample size of fewer than 20, researchers recommend the use of Hedges’ d (Hedges and Olkin, 1985) as an indicator of effect value (Nakagawa and Cuthill, 2007). The formula is as follows:

$$Hedges^{\prime }d=g\left[1-\frac{3}{4\left(n_{1}+n_{2}-2\right)-1}\right]$$

in which

$$g=\frac{{\bar{X}}_{1}-{\bar{X}}_{2}}{s_{pooled}}$$

in which

$$s_{pooled}=\sqrt{\frac{\left(n_{1}-1\right)s_{1}^{2}+\left(n_{2}-1\right)s_{2}^{2}}{n_{1}+n_{2}-2}}$$

Data were processed and analyzed using SPSS 22.0.

## Results

### Comparison of Changes in Forgiveness Level

Table 3 indicates that there was a significant difference between the intervention group and the control group in regard to their changes of ordinal scores on EFI and TRIM-12 from pretest to posttest [EFI: *U*(8, 10) = 17.00, *p* < 0.05, *d* = 1.06; TRIM-12: *U*(8, 10) = 16.50, *p* < 0.05, *d* = −1.24.] As for the dimensions of EFI, there were significant differences between the groups in regard to their changes of ordinal scores of EFI’s NA and NC, although there was no significant difference for positive emotion and cognition. There was a significant difference between the groups in regard to their changes of ordinal scores of EFI’s PB. As no significant differences had been found in EFI’s negative behavior–revenge and NB as shown in Table 3, similar dimensions of “avoidance” and “revenge” in TRIM-12 showed significant differences between the two groups. Thus, on the whole, adolescents with high trait anger in the FI group performed better than did those in the control group, with a mean rank difference equal to 5.18 and −5.29, respectively, in EFI and TRIM-12. Thus, the first hypothesis of this study—FI can help trait-anger adolescents enhance the level of forgiveness toward a particular offender—is supported.

**TABLE 3:** Mann-Whitney *U*-test comparison of the effect of forgiveness intervention on EFI in different subjects.

Variables	M (Mean Rank)	U	ΣN	*p*	Hedges’ *d*
EFI total scores_d		17.00	18	0.041*	1.06
Intervention group	12.38				
Control group	7.20				
-Positive emotion and cognition_d		23.00	18	0.130	0.86
Intervention group	11.63				
Control group	7.80				
-Negative affect_d		12.00	18	0.013*	1.02
Intervention group	13.00				
Control group	6.70				
-Negative cognition_d		18.00	18	0.049*	0.97
Intervention group	12.25				
Control group	7.30				
-Negative behavior_d -Revenge^a^		22.00	18	0.102	0.73
Intervention group	11.75				
Control group	7.70				
-Negative Behavior_d -Avoidance^a^		21.00	18	0.090	0.76
Intervention group	11.88				
Control group	7.60				
-Positive behavior_d		16.50	18	0.036*	1.20
Intervention group	12.44				
Control group	7.15				
TRIM-12 unforgiveness_d		16.50	18	0.036*	−1.24
Intervention group	6.56				
Control group	11.85				
-Avoidance^a^_d		14.00	18	0.020*	−1.18
Intervention group	6.25				
Control group	12.10				
-Revenge^a^_d		20.00	18	0.072^Δ^	−1.04
Intervention group	7.00				
Control group	11.50				

^*a*^The “revenge” and “avoidance” factors of EFI had been reversely scored, reflecting the tendency same as “forgiveness.” While what TRIM-12 measured was the opposite of “forgiveness.” The scale score decreased with the intervention. *p < 0.05; ^Δ^ p < 0.1, marginal significant.

### Comparison of Harmony and Empathy Levels

As shown in Table 4, Mann–Whitney *U*-test showed that there were significant differences between the groups in regard to their changes of ordinal scores on harmony and IRI-C–empathy concern from pretest to posttest [Harmony: *U*(8, 10) = 11.50, *p* < 0.05, *d* = 1.46; IRI-C–empathy concern: *U*(8, 10) = 5.50, *p* < 0.01, *d* = −1.86]. On the whole, adolescents with high trait anger in the FI group changed more than did those in control group, with a Mean Rank difference equal to 6.41 and 7.76, respectively, in Harmony and IRI-C–empathy concern. Although there was only a marginally significant difference on Interpersonal Sensitivity, the *D* value was greater than 0.8. The difference in “perspective taking” was not significant. Despite this, the second hypothesis of this study—FI can improve trait-anger adolescents’ level of empathy and harmony—has been almost validated.

**TABLE 4:** Mann-Whitney *U*-test comparison of the effect of forgiveness intervention on harmony and empathy in different subjects.

Variables	M (mean rank)	U	ΣN	*p*	Hedges’ *d*
Harmony_d		11.50	18	0.011*	1.46
Intervention group	13.06				
Control group	6.65				
Interpersonal sensitivity_d		20.00	18	0.073^Δ^	0.86
Intervention group	12.00				
Control group	7.50				
IRI-C-Perspective-taking_d		38.50	18	0.883	−0.12
Intervention group	9.69				
Control group	9.35				
IRI-C-Empathy Concern_d		5.50	18	0.001**	1.86
Intervention group	13.81				
Control group	6.05				

*P < 0.05; **p < 0.01; ^Δ^p < 0.1, marginal significant.

### Comparison of Changes in Anger Level

Mann–Whitney *U*-test indicated that there was no significant difference between the intervention group and the control group for all the anger outcomes, while in the change of some dimensions of trait anger and the total state anger and its speech/action dimension, the effect values were greater than 0.5, reaching the middle levels (Table 5). Therefore, although the third hypothesis of this study—FI can decrease trait-anger adolescents’ tendency to anger—has not been verified, it is necessary to expand the sample to further verify the role of FI regarding anger-related variables.

**TABLE 5:** Mann-Whitney U-*T*est comparison of the effect of forgiveness intervention on anger in different subjects.

Variables	M (Mean Rank)	*U*	ΣN	*P*	Hedges’ *d*
Trait anger_d		31.00	18	0.421	−0.26
Intervention group	8.38				
Control group	10.40				
-Anger temperament_d		25.50	18	0.188	−0.59
Intervention group	7.69				
Control group	10.95				
-Anger reaction_d		32.00	18	0.462	0.52
Intervention group	10.50				
Control group	8.70				
State Anger_d		22.50	18	0.115	−0.60
Intervention group	7.31				
Control group	11.25				
-Anger Affect_d		35.00	18	0.641	−0.08
Intervention group	8.88				
Control group	10.00				
-Speech/action_d		23.00	18	0.106	−0.78
Intervention group	7.38				
Control group	11.20				
-Anger unleash_d		36.50	18	0.738	−0.17
Intervention group	9.06				
Control group	9.85				

**FIGURE 1:**
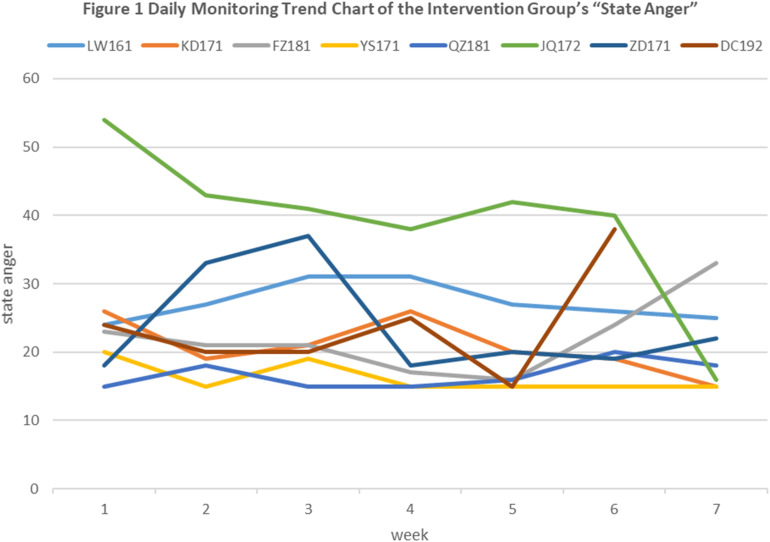
Daily Monitoring Trend Chart of the Intervention Group’s “State Anger.”

As for the dynamic changes of the state anger that were recorded every day, we combined 7 days’ worth of SAS replies and considered them as one unit, and the curve of the overall state anger of the intervention group was drawn (Figure 1). In general, the state anger level of the intervention group showed a slight downward trend.

## Discussion

German positive psychotherapist Nossrat Peseschkian made a classical analogy: if a person’s left leg is lame, in addition to training his left leg to restore its function, he can also train his right leg to strengthen it to compensate for the lost function of his left leg. This principle is very close to the idea of tai chi in Chinese traditional culture. Yin and yang can be transformed into each other. When yin occupies the dominant position, yang is naturally weakened, and once yang increases, the dominant position of yin will naturally decrease (Cui, 2009). The FI in this study showed a similar positive effect on trait-anger adolescents.

### The Influence of FI on Forgiveness

FI has been found to have an effect on common people (e.g., Hui and Chau, 2009; Ji et al., 2016), whereas few studies have used such approach among individuals with high trait anger except Gambaro’s pioneering work, which helped five people with high trait anger forgive (Gambaro, 2002). This present study, according to the forgiveness scales (EFI and TRIM-12), confirms Gambaro’s result with a significant increase in the level of forgiveness toward the offenders among the intervention group, although it was a hard work. Researchers have found that the higher the level of trait anger, the less likely it is to forgive others (Macaskill, 2012; Luo et al., 2013). For these, people experience more hostility and higher stress levels (Maan Diong et al., 2005). The current study suggests that FI plays an effective role in helping high-trait-anger adolescents reduce negative attitudes and increase positive attitudes toward offenders, without which it may take a long period of time. Time is generally regarded as good medicine for healing. Some studies have indeed shown that the degree of forgiveness increases with time (McCullough et al., 2003). However, Mann–Whitney *U*-test for the intervention and control groups revealed that the intervention group showed more increase than the control group did, with a mean rank difference from 4.5 to 6.3 in EFI and its dimensions as well as TRIM-12 and its dimensions. What’s more, FI effectively helped the individual stop accumulating negative emotions with a mean rank difference equal to 6.3 in EFI’s NA dimension. This result is also in line with Pronk et al. (2010), who demonstrated that the degree of forgiveness would increase over time only for people who had high executive functioning. In other words, it may be difficult for some people to forgive only through time, so more external intervention is needed for those difficult ones. We are not to say that trait anger is equivalent to low executive function, but to say that our study suggests that while forgiving a more serious injury event is difficult, FI can help precisely those who have difficulty in forgiving.

### The Influence of FI on Trait Anger

Harris et al. (2006) found FI could decrease trait anger after 6-week sessions among common adults who had experienced a hurtful interpersonal transgression, whereas Rye et al. (2005) did not find the same result in trait anger after 8-week sessions among divorced individuals, neither did Feng et al. (2018) among Chinese angry bus drivers. This suggests that FI is likely to have different effects on reducing trait anger among different people. In the present study, we captured a slight downward trend in state anger level of the intervention group, but non-significant change in trait anger based on the changes in the TAS. Possible reasons might be that (1) trait anger is more difficult to change in trait-anger adolescents than common people; (2) changes in trait anger are difficult to elucidate from statistical data with fewer than 2 months of intervention. The formation of trait anger is the accumulation of years of adverse experiences, which needs continuous FI toward different offender and offense. The FI may have a long-term effect on trait anger, which is different from a general suppression of anger in that, although individuals may reduce their explicit anger tendencies through suppression in the short run, they do not get rid of their internal desire for revenge and have only a limited effect in terms of altering their emotional experiences (Gross and Thompson, 2007), while forgiveness changes the intrinsic motivation of a person (McCullough et al., 2003), and therefore, sufficient time is needed to achieve this goal (Finkel et al., 2002; Fincham et al., 2006). Probably, once changed, it will last long. Harris’s study on an FI for 259 adults who had been subject to severe aggression showed that the training produced improvements in trait anger at 6-week posttest and even at 4-month follow-up (Harris et al., 2006).

In the current study, the daily record of “state anger” showed that the state of persistent anger had decreased. This, at the very least, suggests that FIs for trait-anger adolescents provide an effective way to decrease their anger. Even though forgiveness does not directly affect anger itself, it is still important for character formation. At this point, time is a very important factor. In addition, the rebound in the anger level of some subjects in this study might be related to sudden offensive events in their environment.

### The Influence of FI on Empathy and Harmony

Empathy is one of the indicators that are considered closely related to mental health (Carnicer and Calderon, 2014; Khajeha et al., 2014). It promotes a satisfying connection between people, helps increase altruistic behavior, and reduces aggressive behavior (Carlo et al., 1999; Bjorkqvist et al., 2000; Carnicer and Calderon, 2014). All FI programs consider raising the victims’ empathy level a necessary step, but few researchers have evaluated whether participants’ empathy levels have actually improved from the implementation of FIs.

In this study, based on the scale data of “Interpersonal Sensitivity” and “IRI-C–empathy concern,” the intervention led participants to be more keenly aware of the thoughts and feelings of others and to be more willing and able to sympathize and care for others, increasing their level of empathy (especially emotional empathy). A previous study has shown that the higher the level of empathy, the more forgiving the individual (Macaskill et al., 2002). Our study also supported this finding from an intervention perspective, as increasing empathy levels correlated with an increase in the level of forgiveness.

Psychological harmony is also an important sign of mental health (Nie et al., 2015). The harmony of a person’s inner mind or their interpersonal relationships is an important aspect of psychological harmony. A person with a good state of mental harmony will have characteristics such as high satisfaction with life and work; optimistic, positive, and open-minded personality; fewer negative emotional experiences; good family relationships and interpersonal relationships; more social support; and so on (Research Project Group of Psychological Harmony, 2008). These are characteristics that individuals with high trait anger often lack. Cheung et al. (2005) compiled the CPAI-A, in which “harmony” was used to measure the degree of harmony in the individual’s personality. This concept combined the two aspects of inner harmony and interpersonal harmony (Cheung et al., 2005; Fu, 2006).

The data of the “Harmony Scale” in this study reflected that FI makes the interpersonal and inner aspects of an intervention group more harmonious. This is of great significance for high-trait-anger adolescents. At the beginning of the intervention, several members expressed their hopes that by participating in the group, they would become more peaceful and would learn to control their emotions. If a person can become more peaceful and harmonious by increasing their positive power, this will also play a positive role in their ability to manage irritability, and it will become a protective factor for them in the face of negative life events.

### Limitations and Implications

These findings should be interpreted with caution, mainly because of the small sample size. To compensate, we adopted Hedges’ *d*, which is suitable for small sample effect-size estimation. Although the FI in this study did not change the trait anger in adolescents as significantly as expected, the total and speech/action dimensions of state anger—as well as the temperament and reaction dimensions of trait anger—had moderate effect sizes, and the downward trend can also be seen intuitively in the dynamic evaluation chart of state anger. All of this suggests that the intervention is likely to have some effect, and so, it will be necessary to expand the sample size in a follow-up study.

Although in this study the effects of FI were of good size on interpersonal forgiveness, empathy, and harmony, all effects were measured through self-reported outcomes. The impact of FI on actual behavior and long-term attitudes to the offender were not measured. In addition, like most interventions, the internal validity of the study was high, but external validity may not be high. This intervention took place in a highly controlled setting at a juvenile correctional facility.

The present study was also influenced by other circumstances. For example, at the time the study was carried out, at least two of the subjects were soon to reach the end of their sentence, as a result of which we were unable to follow up with them. The daily environment of the juvenile correctional facility often involved many unexpected conflicts, which was another direct factor that may have impacted our results. This suggests that future studies need to increase the number and longevity of interventions and explore more effective research methods, exploring the optimal frequency at which interventions can be effective and consolidated.

We also produced some novel results. FIs have effectively improved trait-anger adolescents’ levels of forgiveness, harmony, and empathy. This further suggests that the FI for trait-anger adolescents provides an effective way to improve their personality. Even if FIs cannot directly affect anger itself during a short period of time, they can still influence the development of personality as a whole. Therefore, future research needs to explore the psychological mechanism of FIs in trait-anger adolescents. If so, it is not only an effective way of RJ, but also an effective way to prevent crime.

## Conclusion

The conclusions of this study are as follows: (1) the FI significantly improved the level of forgiveness toward specific offenders for trait-anger adolescents; (2) there was no significant increase of the level of trait anger with participants after the intervention, while there was a tendency for improvement, which should be explored further in future research; and (3) the trait-anger adolescents showed an increase in the levels of empathy and harmony after the FI, and these aspects could be protective factors for individuals coping with stressful situations in the future.

## Data Availability Statement

The raw data supporting the conclusions of this article will be made available by the authors, without undue reservation.

## Ethics Statement

The studies involving human participants were reviewed and approved by the Ethics Review Committee of Nanjing Normal University. Written informed consent to participate in this study was provided by the participants’ legal guardian/next of kin.

## Author Contributions

LT was responsible for the study design, implementation, analysis, interpretation of data, drafting the work and revising it critically for important intellectual content, and reprocesses the data as revising the manuscript. MJ participated the study design, interpretation of data, and revision the draft. TZ participated in the whole process of writing this manuscript, working hard with the LT on the final revision of the manuscript and responsible for the submission process and at the same time, her project also supported this study, and accountable for all aspects. All authors contributed to manuscript revision, read and approved the submitted version.

## Conflict of Interest

The authors declare that the research was conducted in the absence of any commercial or financial relationships that could be construed as a potential conflict of interest.
